# *Ustilaginoidea virens* secreted effector UvSec117 hijacks OsWRKY31‐OsAOC module to suppress jasmonic acid‐mediated immunity in rice

**DOI:** 10.1111/pbi.14452

**Published:** 2024-08-16

**Authors:** Yuhang Duan, Guogen Yang, Jintian Tang, Yuan Fang, Hailin Wang, Zhaoyun Wang, Hao Liu, Xiaolin Chen, Junbin Huang, Jing Chen, Qiutao Xu, Lu Zheng, Xiaoyang Chen

**Affiliations:** ^1^ Anhui Province Key Laboratory of Crop Integrated Pest Management Anhui Agricultural University Hefei China; ^2^ The Key Lab of Plant Pathology of Hubei Province/State Key Laboratory of Agricultural Microbiology Huazhong Agricultural University Wuhan China; ^3^ Zhejiang Provincial Key Laboratory of Biometrology and Inspection & Quarantine, College of Life Sciences China Jiliang University Hangzhou China; ^4^ State Key Laboratory for Conservation and Utilization of Subtropical Agro‐bioresources Guangxi University Nanning China

**Keywords:** Rice false smut, effector, WRKY transcription factor, disease resistance, jasmonic acid

Rice false smut (RFS) caused by *Ustilaginoidea virens* is one of the most important disease in rice (*Oryza sativa*)‐growing regions worldwide. RFS not only causes rice yield losses but also potentially threatens human and animal health by producing cyclopeptide mycotoxins (Sun *et al*., 2020). Introducing genetically encoded resistance is an environmentally friendly, economical approach to controlling plant diseases (Yu *et al*., 2023). However, at present, the varieties and gene resources of resistance to RFS are still extremely scarce, and it is difficult to identify major resistance genes against RFS. Uncovering the functions of the *U. virens* effectors and molecular mechanism of the rice, *U. virens* interaction can help to identify molecular probes for discovering disease resistance‐related genes (Wang and Kawano, 2022).

In previous studies, we identified UvSec117 as a key virulence effector in *U. virens*, and found rice transcription factor OsWRKY31 in a screen for proteins that interact with UvSec117 (Chen *et al*., 2022). WRKY transcription factors have many regulatory roles in development and response to biotic/abiotic stresses in plants (Wang *et al*., 2023). However, little is known about the regulatory functions of WRKY genes in the plant resistance to grain‐infecting pathogens. In this work, we confirmed interactions between UvSec117 and OsWRKY31 in a directed yeast two‐hybrid assay (Figure 1a; Data S1). In a co‐immunoprecipitation (Co‐IP) assay by rice protoplasts transiently co‐expressing *OsWRKY31‐Flag* and *UvSec117‐GFP* constructs, UvSec117 was immunoprecipitated by OsWRKY31 (Figure 1b). In a pull‐down assay using recombinant OsWRKY31‐GST and UvSec117‐His purified from *Escherichia coli*, OsWRKY31‐GST was pulled down by His beads coated with UvSec117‐His (Figure 1c). We also validated the interaction between UvSec117 and OsWRKY31 by a luciferase complementation imaging (LCI) assay in *N. benthamiana* leaves (Figure 1d). When we transiently co‐expressed *UvSec117‐cYFP* and *OsWRKY31‐nYFP* constructs in rice protoplasts and performed a bimolecular fluorescence complementation (BiFC) assay, we detected YFP (yellow fluorescent protein) fluorescence in the nucleus (Figure 1e). Collectively, these results suggest that UvSec117 interacts with OsWRKY31 in vivo and in vitro.

**Figure 1:**
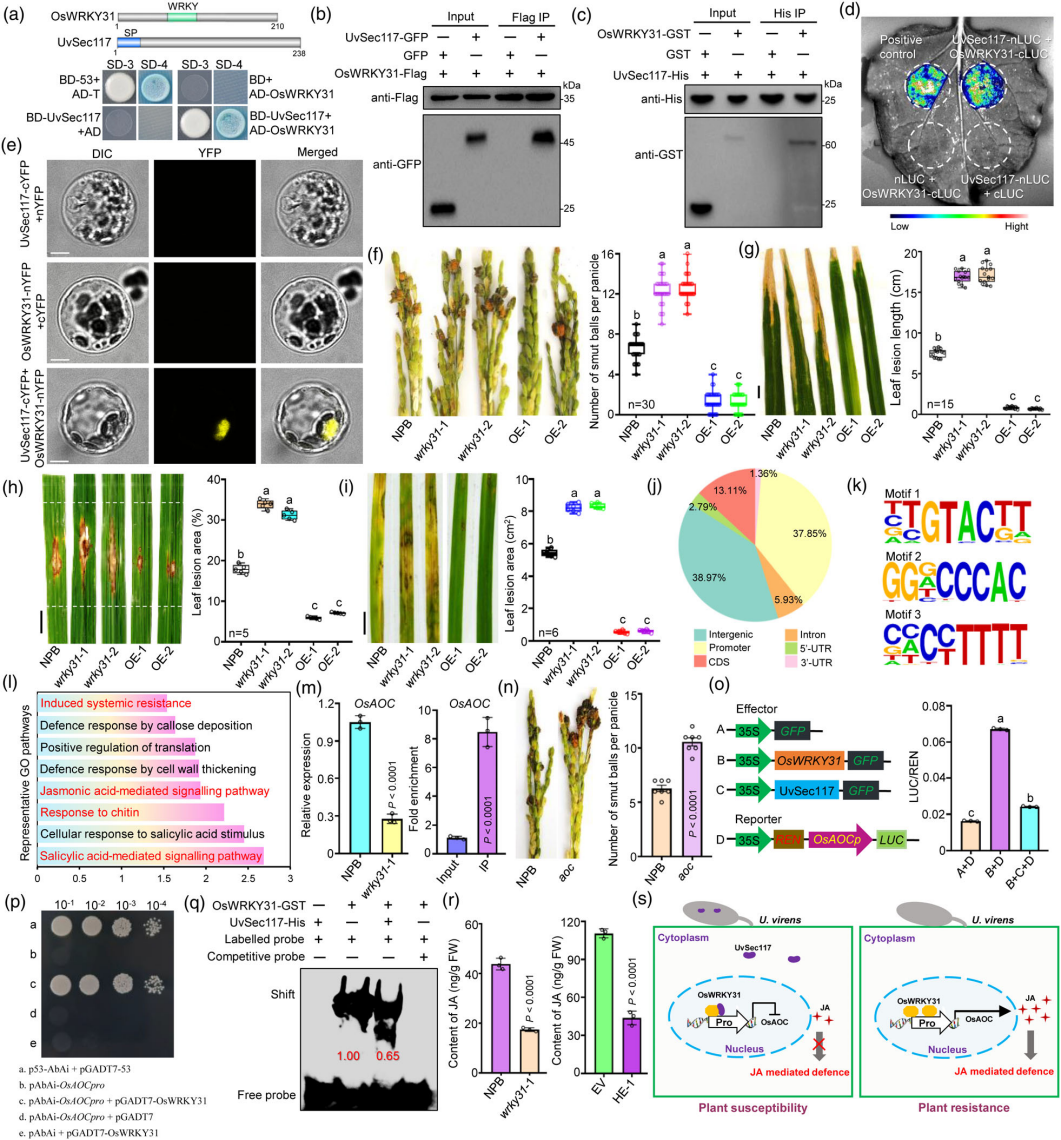
UvSec117 hijacks OsWRKY31‐OsAOC module to suppress jasmonic acid mediated immunity in rice. (a) Yeast two‐hybrid analysis of the interaction between UvSec117 and OsWRKY31. (b) Co‐IP showing that UvSec117 interacts with OsWRKY31 in vivo. (c) GST pull‐down assay to detect the interaction between UvSec117‐His and OsWRKY31‐GST. (d) LCI assay of the interaction between UvSec117‐nLUC and OsWRKY31‐cLUC in *N. benthamiana* epidermal cells. (e) BiFC assay of the interaction between UvSec117 and OsWRKY31; scale bar, 5 μm. (f) Resistance of NPB, *OsWRKY31*‐OE and *wrky31* plants against *U. virens* HWD‐2 at 21 dpi. (g) Disease symptoms and lesion lengths of NPB, *OsWRKY31*‐OE, and *wrky31* plants at 14 dpi with *Xanthomonas oryzae* pv. *oryzae* strain PXO99 when inoculated via the scissor‐clipping method; scale bar, 1 cm. (h) Disease symptoms and leaf lesion areas of NPB, *OsWRKY31*‐OE and *wrky31* plants at 14 dpi following spot inoculation with *Magnaporthe oryzae* strain ZB25; scale bar, 1 cm. (i) Disease symptoms and leaf lesions of NPB, *OsWRKY31*‐OE and *wrky31* plants at 3 dpi with *Rhizoctonia solani* strain HG81; scale bar, 1 cm. (j) OsWRKY31‐binding sites were shown in the genome of rice. (k) Top three OsWRKY31 binding motifs identified using MEME. (l) Representative GO pathways of OsWRKY31‐target genes. (m) RT‐qPCR and ChIP‐qPCR of *OsAOC* expression in NPB and *wrky31*‐1 plants. (n) Resistance of NPB and *OsAOC* mutant plants against *U. virens* HWD‐2 at 21 dpi. (o) UvSec117 inhibits OsWRKY31‐activated *OsAOCpro‐LUC* transcription. *OsAOCpro‐LUC* was infiltrated alone, or together with OsWRKY31 and UvSec117. (p) Yeast one‐hybrid analysis indicated OsWRKY31 can bind to the promoter of *OsAOC*. (q) UvSec117 inhibits the DNA‐binding activity of OsWRKY31. OsWRKY31‐His was incubated with a biotin‐labelled probe within the *OsAOC* promoter and subjected to EMSA. Unlabelled probe was used as the competitor (100×). UvSec117‐His was preincubated with OsWRKY31‐GST for EMSA. (r) JA concentrations in NPB and *wrky31*‐1, EV and HE‐1 rice spikelets. Phytohormones were analysed by liquid chromatography–tandem mass spectrometry. (s) A working model illustrating how UvSec117 manipulates OsWRKY31 to suppress rice immunity during *U. virens* infection. Data are means ± SD (*n* = 3 unless otherwise indicated). The *P*‐values were determined by unpaired *t*‐tests and Tukey's multiple comparisons test.

To explore the role of OsWRKY31 in resistance against RFS fungus or other rice pathogens, we generated *OsWRKY31* knockout mutant plants (*wrky31*) (Figure S1a) and *OsWRKY31*‐overexpressing transgenic rice lines (*OsWRKY31*‐OE) (Figure S1b). The agronomic traits of *wrky31* and *OsWRKY31*‐OE plants were similar to those of wild‐type *Nipponbare* (NPB) (Figure S1c,d). Following inoculation with different rice pathogens, *OsWRKY31*‐OE plants were less susceptible and *wrky31* plants were more susceptible to the RFS, bacterial blight, rice blast and sheath blight than NPB plants (Figure 1f–i), indicating that OsWRKY31 positively regulates the resistance of rice to multiple diseases.

To identify global targets of the transcription factor OsWRKY31, we performed chromatin immunoprecipitation followed by deep sequencing (ChIP‐seq) using *OsWRKY31*‐OE plants with an anti‐Flag antibody. In total, we identified 4626 peaks (1054 target genes, Data S2). A significant majority (> 60%) of these peaks are located within genic regions, with the modifications being highly enriched at the promoters of protein‐coding genes (Figure 1j). MEME (Multiple EM for Motif Elicitation) analysis revealed that most OsWRKY31‐bound DNA motifs contained the sequence TTGTACTT, GGGCCCAC or CCCCTTTT (Figure 1k). Gene ontology (GO) analysis revealed that the target genes were enriched for induced systemic resistance and salicylic acid (SA)/jasmonic acid (JA)‐mediated signalling pathways (Figure 1l). RT‐qPCR showed that the key JA biosynthesis gene *OsAOC* (*ALLENE OXIDE CYCLASE*) is significantly downregulated in *wrky31*‐1 plants, and ChIP‐qPCR confirmed that OsWRKY31 binds to the *OsAOC* promoter (Figure 1m). Knockout of *OsAOC* in rice enhances its susceptibility to RFS (Figure 1n). *OsWRKY31* expression in *N. benthamiana* significantly enhanced firefly luciferase (LUC) activity derived from the *OsAOCpro*‐*LUC* reporter. Co‐infiltration of *UvSec117* with *OsAOCpro*‐*LUC* inhibited OsWRKY31‐induced LUC activity, whereas co‐infiltration of *GFP*, did not (Figure 1o). Yeast one‐hybrid results showed that OsWRKY31 can bind the promoter of *OsAOC* (Figure 1p). In an electrophoretic mobility shift assay (EMSA) assay, OsWRKY31‐His specifically bound to the *OsAOC* promoter; addition of unlabelled competitive probe decreased this binding. Preincubation with UvSec117 reduced the DNA‐binding activity of OsWRKY31 (Figure 1q), indicating that UvSec117 directly inhibits the DNA‐binding activity of OsWRKY31. Moreover, the contents of JA were significantly lower in *wrky31*‐1 than in NPB rice spikelets; in HE‐1 (Heterologous expression of *UvSec117* transgenic plants) relative to EV (empty vector transgenic plants) rice spikelets (Figure 1r). These results indicate that OsWRKY31 regulating the JA‐mediated defence was suppressed by UvSec117.

In this study, we found that the transcription factor OsWRKY31 functions as a key positive regulator to broad‐spectrum disease resistance. Here, we provide a comprehensive genome‐wide binding map of OsWRKY31 and its regulatory network, and further describe a previously unknown regulatory role where OsWRKY31 mediates the JA‐mediated signalling pathway to regulate plant immunity. Collectively, this study unveils a pivotal virulence strategy employed by *U. virens*, the secretory effector UvSec117 inhibits OsWRKY31 binding to target gene promoters like *OsAOC*, thereby suppressing JA‐mediated defence (Figure 1s). Moreover, this investigation highlights the critical role of OsWRKY31 as a crucial component in orchestrating multi‐pathogen resistance, further underscoring its significance in plant defence mechanisms. The *OsWRKY31*‐OE lines generated in this study may provide valuable germplasm resources for rice disease resistance breeding, which has important theoretical and practical value.

## Conflict of interest

The authors declare no conflict of interest.

## Author contributions

Y.D. J.T. and G.Y. performed most of the experiments. Q.X. performed the data analyses. L.Z., H.L., H.W., Z.W., Y.F., J.H., J.C. and X‐L.C. provided technical support. X‐Y.C. and L.Z. wrote and revised the manuscript. All authors have read and approved the final manuscript.

## Supporting information


**Data S1** Materials and methods.

**Data S2** ChIP‐seq data statistics of the OsWRKY31.

**Figure S1** OsWRKY31 transgenic rice plants were achieved without adverse effects on plant growth or yield. (a) Mutations identified within sgRNA target sites of *OsWRKY31* in rice generated by CRISPR/Cas9‐mediated genome editing. (b) RT‐qPCR of *OsWRKY31* expression in NPB and *OsWRKY31*‐OE transgenic rice plants. (c, d) Morphology and agronomic traits of mature wild‐type NPB, *OsWRKY31*‐OE and *wrky31* plants grown in the field. Data are means ± SD (*n* = 3 unless otherwise indicated). The *P*‐values were determined by Tukey's multiple comparison tests compared to NPB.

## Data Availability

The data that support the findings of this study are available on request from the corresponding author. The data are not publicly available due to privacy or ethical restrictions.
