# Supplementary material for: Ustilaginoidea virens secreted effector UvSec117 hijacks OsWRKY31‐OsAOC module to suppress jasmonic acid‐mediated immunity in rice
Source: Plant Biotechnol J. 2024 Aug 16;22(12):3342–4. doi: 10.1111/pbi.14452 (PMC11606405; doi:10.1111/pbi.14452)
Supplement: Supplementary file 1 — Data S1 Materials and methods. [file PBI-22-3342-s003.doc]

**Materials and Methods**

**Plant materials, strains and inoculation assays**

*Oryza sativa* cvs. ‘Nipponbare’ (NPB), and *Nicotiana benthamiana* plants were grown in a glasshouse. The full-length coding sequence of *OsWRKY31* was cloned into the overexpression vector pU1301-Flag with the CaMV 35S promoter. The sgRNA (GCTGCAGACTGCATCTGGGAAGG) of *OsWRKY31* was cloned into the CRISPR/Cas9 vector pRGEB32. Transgenic rice (*Oryza sativa*) plants were generated in the Nipponbare (NPB) background, and rice transformation was performed by Wuhan Tianwen Biotechnology Co., Ltd (Wuhan, China). Rice plants were inoculated with the fungal strains *Magnaporthe oryzae* strain ZB25, *Xanthomonas oryzae* pv. *oryzae* (*Xoo*) strain PXO99, *Rhizoctonia solani* strain HG81, and *U. virens* HWD-2.

Rice plants at the booting stage were inoculated with *U. virens* HWD-2 mycelial/spore suspensions (1×10^6^ conidia/mL) using a syringe, and the number of false smut balls was counted 21 days post inoculation (dpi) (Chen *et al.,* 2022). For spot-inoculations, the leaves of 4-week-old plants were punctured with a needle, and a droplet of 10 μL *M. oryzae* ZB25 conidial suspension (1×10^5^ conidia/mL) was placed at each wound site, and the disease lesions were examined at 14 dpi (Chen *et al.,* 2023). Rice plants were inoculated with *Xoo* PXO99 at the booting stage using the leaf clipping method, and the lesion lengths were scored at 14 dpi (Chen et al., 2022). The leaves of four-week-old rice plants were inoculated with *R. solani* HG81, and the lesion areas were scored at 3 dpi and calculated using ImageJ software. All inoculation experiments were repeated three times.

Protoplast transfection was performed using the polyethylene glycol (PEG)-mediated method (Wang et al., 2024). The plasmids and protoplasts were mixed, and the same volume of PEG solution (0.6 M D-mannitol, 100 mM CaCl_2_, and 40% [w/v] PEG 4000) was added. The mixture was incubated at 28 ºC for 15 min, and W5 was added to terminate the reaction. The protoplasts were collected by centrifugation and resuspended in W5. Following incubation at 28ºC in the dark for 16-24 h, the protoplasts were used for assay.

**Yeast one-hybrid (Y1H) assay**

The *OsWRKY31* cDNA sequence was ligated to pGADT7 as the prey, and approximately 50-bp sequence of the promoter region of *OsAOC* from ChIP-seq was ligated to the pAbAi vector (Clontech, USA) as a bait. The pAbAi-*OsAOCpro* vector was transformed into Y1HGold (Clontech, USA) cells, and transformed strains were isolated with SD/-Ura medium and confirmed by PCR. Then, the pGADT7-OsWRKY31 vector was transformed into Y1HGold cells harboring the pAbAi-*OsAOCpro* vector, and the transformants were isolated with SD/-Ura media with 700 ng/ml Aureobasidin A (AbA). The p53-AbAi and pGADT7-53 vectors co-transformants were used as the positive control, pAbAi-*OsAOCpro* and pGADT7, pAbAi and pGADT-OsWRKY31 vector co-transformants as the negative control.

**Yeast two-hybrid (Y2H) assay**

The cDNA sequence of *UvSec117* (without the SP) and *OsWRKY31* were ligated with pGBKT7 and pGADT7, respectively. For interaction analysis of UvSec117 and OsWRKY31 in yeast, the vectors pGBKT7-*UvSec117* and pGADT7-*OsWRKY31*, pGBKT7-*UvSec117* and pGADT7, or pGBKT7 and pGADT7-*OsWRKY31* were co-transformed into the Y2H Gold strain. Positive clones were selected on SD–Trp–Leu–His medium, and further confirmed on SD–Trp–Leu–His–Ade medium containing X-α-Gal. The interaction between BD-53 and AD-T was used as the positive control, and the pairs BD and AD-OsWRKY31, BD-UvSec117 and AD were used as negative controls. SD–3, synthetic defined medium SD–Trp–Leu–His; SD–4, SD–Trp–Leu–His–Ade; BD, pGBKT7; AD, pGADT7.

**Co-immunoprecipitation (Co-IP) assays**

The cDNA sequences of *UvSec117* and *OsWRKY31* were ligated with the vectors pCNG (GFP tags) and pCNF (Flag tags) and then introduced individually into Agrobacterium strain GV3101. Agrobacterium carrying the pCNG-*UvSec117* and pCNF-*OsWRKY31* vectors were co-infiltrated into rice protoplasts. Total proteins were extracted from rice protoplasts and incubated with Anti-Flag M2 affinity gel (Yeasen Biotech, Shanghai, China). Proteins eluted from gels were analyzed by immunoblotting with anti-GFP or anti-Flag antibodies (ABclonal, Wuhan, China).

**GST pull-down assays**

The cDNA sequences of *UvSec117* (without the SP) and *OsWRKY31* were ligated with the vectors pET28a (His tags) and pGEX-4T-2 (GST tags) and then introduced individually into *Escherichia coli* BL21(DE3) cells. The UvSec117-His fusion protein was extracted from *E. coli* cells and incubated with 100 µL His beads (Yeasen Biotech, Shanghai, China) at 4°C for 4 h with shaking. After centrifugation at 4°C, the beads were collected and washed with phosphate buffered saline (PBS) three times. Beads were then incubated with recombinant OsWRKY31-GST protein at 4°C for 2 h with shaking and then washed with PBS three times. Beads were boiled for 5 min at 100°C in 40 µL SDS sample loading buffer, and the proteins were analyzed by immunoblotting with anti-His and anti-GST antibodies (ABclonal, Wuhan, China).

**Firefly luciferase complementation imaging (LCI) assay**

The coding sequences of *UvSec117* was individually ligated into the vector pCAMBIA1301-nLuc, and the coding sequence of *OsWRKY31* was ligated into the vector pCAMBIA1301-cLuc. Agrobacterium strain GV3101 cells were transformed individually with *UvSec117*-nLuc, *OsWRKY31*-cLuc, -nLuc, or -cLuc, the appropriate combination of cultures was resuspended in infiltration buffer to a final OD600 of 1.6, mixed in 1:1 ratio (v/v) and then infiltrated into the leaves of *N. benthamiana* plants. At 2 d after infiltration, *N. benthamiana* leaves were infiltrated with 1 mM luciferin (Gold Biotechnology, USA). Bioluminescence images were taken with a Chemi-Image System (Tanon 5200Multi, China).

**Bimolecular fluorescence complementation** **(BiFC) assays**

The cDNA sequences of *UvSec117* and *OsWRKY31* were ligated with the BiFC vectors pCAMBIA1301-nYFP and pCAMBIA1301-cYFP, respectively. The resulting constructs encoding the UvSec117-cYFP and OsWRKY31-nYFP fusion proteins were co-infiltrated into rice protoplasts, and the fluorescence signal of the infiltrated areas was analyzed with a LSCM.

**Immunoblot analysis**

Proteins were separated by 12% SDS-PAGE and transferred onto a polyvinylidene fluoride (PVDF) membrane (Merck Millipore, Burlington, MA, USA) using wet transfer at 80 V for 90 min with a BioRad electroblotting apparatus. Membranes were blocked in Tris buffered saline with 0.1% Tween 20 (TBST) containing 5% (w/v) non-fat dry milk at room temperature for 2 h. Primary antibodies used were anti-GFP, anti-Flag, anti-His, or anti-GST (ABclonal, Wuhan, China). The membrane was incubated with primary antibodies in TBST with 5% non-fat dry milk at room temperature for 2 h with shaking and washed six times (5 min each) with TBST. Next, the membrane was incubated with goat anti-mouse (ABclonal, Wuhan, China) secondary antibody in TBST with 5% non-fat dry milk at room temperature for 1.5 h with shaking. The membrane was washed six times (5 min each) with TBST, and the signals were detected using Pierce ECL Western blotting substrate (Thermo Fisher Scientific, USA) in a ChemiDoc XRS+ system (Bio-Rad).

**ChIP-seq and data analysis**

Chromatin immunoprecipitation (ChIP) was performed as described previously (Lu et al., 2018). Briefly, 5 g of *OsWRKY31-*OE rice spikelets was crosslinked in 1% (v/v) formaldehyde. Chromatin was extracted and fragmented to 100- to 500-bp fragments by sonication, and ChIP was performed using the anti-Flag antibody (Abcam，ab1162). Sequencing libraries were constructed with DNA from ChIP following the Illumina TruSeqCHIP Sample Prep Set A protocol and sequenced on an Illumina HiSeq2000 as 150-bp paired-end reads by Wuhan IGENEBOOK Biotechnology Co., Ltd. The precipitated and input DNA was analyzed by ChIP-qPCR. At least three biological replicates were tested per treatment.

FastP (v0.232) was used with the default parameters to trim low-quality bases and adapters. Bowtie2 (version 2.3.5.1) was used to map cleaned reads to the rice genome (MSU7.0) using the default settings (Langmead and Salzberg, 2012). Samtools (v1.9) was then used to remove duplicate reads (*L*i et a*l.*, 2009). Peaks were annotated by Homer (v4.11) using the default parameters (Heinz et al., 2010). Metaplots were generated by ngsplot (v2.61) (Sh*e*n et a*l*., 2014). Chromosome plots, correlation plots, and scatter plots were generated in R (v3.5). GO pathways were analyzed by clusterProfiler (v4.0) (*W*u et al*.*, 2021).

**Protein purification and EMSA**

The pGEX-4T-2-*OsWRKY31* and pET-28a-*UvSec117* vectors were transformed into BL21 (DE3) strain. Tthese strains were cultured in 200 mL LB medium in shake flasks (180 rpm) at 37°C for 12 h. Then, the cells were induced with 0.1 M IPTG at 28°C for 8 h and harvested. The cells were washed with PBS buffer and disrupted with ultrasound to extract protein. The UvSec117-His and OsWRKY31-GST proteins were purified with HisPur™ Ni-NTA Resin and GSTrap^TM^ Resin (Thermo Scientific, USA), respectively. Total 1 mL GSTrap or HisPur™ Ni-NTA was added to the chromatography column, and washed three times with 500 µL PBS. The extracted protein solution was passed through the column, washed three times with PBS, and finally eluted with the eluent buffer (50 mM Tris-HCl, 10 mM GST).

Electromobility shift assays (EMSA) were conducted using a LightShift Chemiluminescent EMSA kit (Thermo, Rockford). Binding reactions were carried out in a 20 µL volume of reaction buffer (2 µL 10× Binding Buffer, 1 µL 50% Glycerol, 1 µL 100 mM KCl, 1% NP-40, 1 µL/µL poly[dI-dC]) for 20 min at room temperature. Labeled DNA probe (ATTTAACCAGGGTTCGGCCCTTTGGTTAGGTGGGAC) was incubated with OsWRKY31-GST, 100x unlabeled DNA probe was used for competition. UvSec117-His was preincubated with OsWRKY31-GST for 30 min at room temperature before DNA binding. The reaction was stopped by adding DNA loading buffer and the samples were separated by a 6% native PAGE gel. After electrophoresis, the gel was autoradiographed.

**JA measurement**

For jasmonic acid (JA) metabolites measurements, the NPB and *wrky31*-1, EV and HE-1 rice spikelets were separately collected and freeze-dried. The freeze-dried tissues were used for metabolites measurement was performed as described previously (Xu et al., 2023). The sample extracts were analyzed using an LC–ESI–MS/MS system (HPLC, Shim-pack UFLC Shimadzu CBM20A system).

**Dual luciferase reporter assay**

The dual luciferase reporter assay was performed as described previously (Yang et al., 2022). The promoter sequence of *OsAOC* was ligated with the reporter vector pGreen-LUC. The cDNA sequences of *UvSec117* and *OsWRKY31* were ligated with the vectors pCNG. These constructs were individually transformed into Agrobacterium strain GV3101. Agrobacterium cultures containing these reporter and effector vectors were co-infiltrated into *N. benthamiana* leaves. Three days after infiltration, the ratio of LUC to Ren activity was measured using the Dual-Glo® Luciferase Assay System (Promega) on an Infinite M200 luminometer (Tecan, Mannerdorf, Switzerland).

**RT-qPCR and ChIP-qPCR analysis**

Total RNA was extracted using TRIzol reagent (Vazyme Biotech, Nanjing, China). First-strand cDNA synthesis was carried out with a cDNA Synthesis SuperMix (TransGen Biotech, Beijing, China). Quantitative reverse-transcription (qRT)-PCR was performed with TransStart® Tip Green qPCR SuperMix (TransGen Biotech). Transcript levels of the rice ubiquitin gene (*OsUBQ1*) and Input were used for normalization in RT-qPCR and ChIP-qPCR, respectively. At least three biological replicates were tested per treatment.

**Statistical analyses**

The data are presented as means ± standard deviation (SD). Statistical analysis was performed using Student’s *t*-test (paired, two-sided) for comparing two groups, and one-way analysis of variance (ANOVA) with Tukey’s multiple comparison tests for comparing three or more groups. *P* < 0.05 was considered as statistically significant. Data analysis was conducted using GraphPad Prism Software (Version 8.0.1).

**References**

Chen, X.Y., Li, X.B., Duan, Y.H., Pei, Z.X., Liu, H., Yin, W.X., Huang, J.B., Luo, C.X., Chen, X.L., Li, G.T., Xie, K.B., Hsiang, T., and Zheng, L. (2022) A secreted fungal subtilase interferes with rice immunity via degradation of SUPPRESSOR OF G2 ALLELE OF *skp1*. *Plant Physiol.* **190**(2), 1474–1489.

Chen, X.Y., Xu, Q.T., Yue, Y.P., Duan, Y.H., Liu, H., Chen, X.L., Huang, J.B., and Zheng, L. (2023) Comparative oxidation proteomics analyses suggest redox regulation of cytosolic translation in rice leaves upon *Magnaporthe oryzae* infection. *Plant Commun.* **4**, 100550.

Heinz, S., Benner, C., Spann, N., Bertolino, E., Lin, Y.C., Laslo, P., Cheng, J.X., Murre, C., Singh, H., and Glass, C.K. (2010) Simple combinations of lineage-determining transcription factors prime cis-regulatory elements required for macrophage and B cell identities. *Mol. Cell* **38**(4), 576–589.

Langmead, B., and Salzberg, S.L. (2012) Fast gapped-read alignment with Bowtie 2. *Nat. Methods* **9**(4), 357–359.

Li, H., Handsaker, B., Wysoker, A., Fennell, T., Ruan, J., Homer, N., Marth, G., Abecasis, G., and Durbin, R. (2009) The Sequence Alignment/Map format and SAMtools. *Bioinformatics* **25**(16), 2078–2079.

Lu, Y., Xu, Q.T., Liu, Y., Yu, Y., Cheng, Z.Y., Zhao, Y., and Zhou, D.X. (2018) Dynamics and functional interplay of histone lysine butyrylation, crotonylation, and acetylation in rice under starvation and submergence. *Genome Biol.* **19**, 144.

Shen, L., Shao, N., Liu, X., and Nestler, E. (2014) ngs.plot: Quick mining and visualization of next-generation sequencing data by integrating genomic databases. *BMC Genomics* **15**, 284.

Wang, B., Xue, P., Zhang, Y., Zhan, X., Wu, W., Yu, P., Chen, D., Fu. J., Hong, Y., Shen, X., Sun, L., Cheng, S., Liu, Q., and Cao, L. (2024) OsCPK12 phosphorylates OsCATA and OsCATC to regulate H_2_O_2_ homeostasis and improve oxidative stress tolerance in rice. *Plant Comm.* **5**, 100780.

Wu, T., Hu, E., Xu, S., Chen, M.J., Guo, P.F., Dai, Z.H., Feng, T.Z., Zhou, L., Tang, W.L., Zhan, L., Fu, F.C., Liu, S.S., Bo, X.C., and Yu, G.C. (2021) clusterProfiler 4.0: A universal enrichment tool for interpreting omics data. *Innovation (Camb)* **2**(3), 100141.

Xu, L., Zhao, H.Y., Wang, J.B., Wang, X.M., Jia, X.Q., Wang, L., Xu, Z., Li, R.L., Jiang, K., Chen, Z.X., Luo, J., Xie, X.D., and Yi, K.K. (2023) AIM1-dependent high basal salicylic acid accumulation modulates stomatal aperture in rice. *New Phytol.* **238**, 1420–1430.

Zhang, N., Zhou, S., Yang, D., and Fan, Z. (2020) Revealing shared and distinct genes responding to JA and SA signaling in *Arabidopsis* by meta-analysis. *Front. Plant Sci.* **11**, 908.

Yang, Q.R., Yang, X.P., Wang, L., Zheng, B.B., Cai, Y.M., Ogutu, C.O., Zhao, L., Peng, Q., Liao, L., Zhao, Y., Zhou, H., and Han, Y.P. (2022) Two R2R3-MYB genes cooperatively control trichome development and cuticular wax biosynthesis in *Prunus persica. New Phytol.* **234**, 179–196.
