# Supplementary material for: Ustilaginoidea virens secreted effector UvSec117 hijacks OsWRKY31‐OsAOC module to suppress jasmonic acid‐mediated immunity in rice
Source: Plant Biotechnol J. 2024 Aug 16;22(12):3342–4. doi: 10.1111/pbi.14452 (PMC11606405; doi:10.1111/pbi.14452)
Supplement: Supplementary file 3 — Figure S1 OsWRKY31 transgenic rice plants were achieved without adverse effects on plant growth or yield. (a) Mutations identified within sgRNA target sites of OsWRKY31 in rice generated by CRISPR/Cas9‐mediated genome editing. (b) RT‐qPCR of OsWRKY31 expression in NPB and OsWRKY31‐OE transgenic rice plants. (c, d) Morphology and agronomic traits of mature wild‐type NPB, OsWRKY31‐OE and wrky31 plants grown in the field. Data are means ± SD (n = 3 unless otherwise indicated). The P‐values were determined by Tukey's multiple comparison tests compared to NPB. [file PBI-22-3342-s002.docx]

**(a)** NPB: ACTGCATCTGGGAAGG

**(b)**

*wrky31*-1 : ACTGCATCTGAGGAAGG + 1bp 12


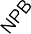

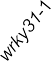

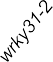

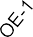

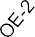

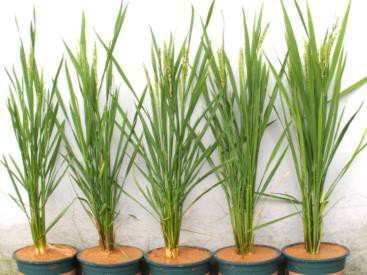

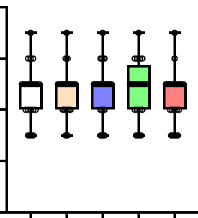

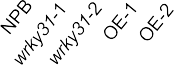

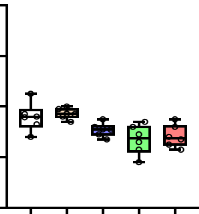

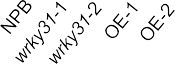


a

**(c)**

a a a a a

22.8

22.6

a

22.4

a

a a a

22.2

n=20

22.0 n=6


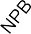

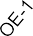

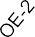

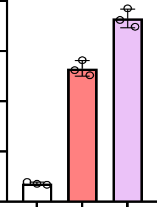


b

c

Relative expression

*wrky31*-2: ACTGCATCT–GGAAGG - 1bp

9


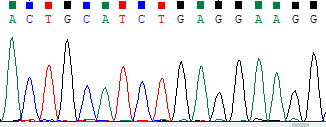


*wrky31*-1

6

3


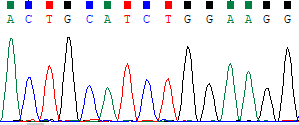


*wrky31*-2

0

**(d)** 94


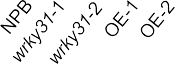

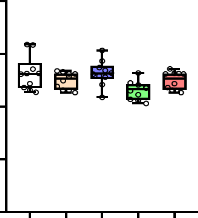


a

a

a a a

n=10

Seed setting rate (%)

92

90

88

86

100 12

10


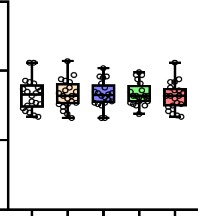

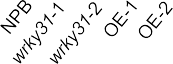


a a

a

a

a

n=20

Plant height (cm)

Effective tillers

Thousand grain weight (g)

95

8

90

6

85 4

**Figure S1 OsWRKY31 transgenic rice plants were achieved without adverse effects on plant growth or yield.** (a) Mutations identified within sgRNA target sites of *OsWRKY31* in rice generated by CRISPR/Cas9-mediated genome editing. (b) RT-qPCR of *OsWRKY31* expression in NPB and *OsWRKY31*-OE transgenic rice plants. (c, d) Morphology and agronomic traits of mature wild-type NPB, *OsWRKY31*-OE, and *wrky31* plants grown in the field. Data are means ± SD (n = 3 unless otherwise indicated). The *P*-values were determined by Tukey’s multiple comparison tests compared to NPB.
